# Decadal Changes in the Abundance and Length of Snapper (*Chrysophrys auratus*) in Subtropical Marine Sanctuaries

**DOI:** 10.1371/journal.pone.0127616

**Published:** 2015-06-10

**Authors:** Hamish A. Malcolm, Arthur L. Schultz, Patrick Sachs, Nicola Johnstone, Alan Jordan

**Affiliations:** 1 Marine Ecosystems Research, NSW Department of Primary Industries, PO Box 4297, Coffs Harbour, NSW, 2450, Australia; 2 Southswell Marine, 204 Schnapper Beach Road, Urunga, NSW, 2455, Australia; 3 Australian Fisheries Management Authority, Box 7051, Canberra, ACT, 2610, Australia; 4 Solitary Islands Marine Park, NSW Department of Primary Industries, PO Box 4297, Coffs Harbour, NSW, 2450, Australia; 5 Marine Ecosystems Research, NSW Department of Primary Industries, Private Bag 1, Nelson Bay, NSW, 2315, Australia; University of Windsor, CANADA

## Abstract

Abundance and length of the highly-targeted snapper Chysophrys auratus were compared between sites in 'no take' areas (Sanctuary Zones: SZ), partial protected areas which are fished (Habitat Protection Zones: HPZ), and areas outside (Outside) the Solitary Islands Marine Park (SIMP), Australia. Baited Remote Underwater Video (BRUV) sampling on shallow rocky reef (15 - 25 m) was conducted annually from 2002 until 2014 in the Austral-winter, covering the decade after these marine park zones were established (2002). Additional deeper sites (25 - 40 m) were sampled in 2010-2011 to assess if findings were more-broadly applicable. Lengths were measured using stereo-BRUVs from 2011-2014. Snapper were significantly more abundant in SZ overall and in most years compared with the other two management types, which did not significantly differ. Snapper rapidly increased after 2 - 3 years protection in all management types, especially SZ. Snapper were present on more SZ deployments than HPZ and Outside after the same period. The positive SZ response in snapper abundance on shallower reef was also found at a broader spatial scale on deeper sites. Again the two fished management types did not show significant differences among each other. There was considerable variation in snapper abundance between years, with strong peaks in 2005, 2009 and 2014 especially in SZ. Abundances remained higher in SZ in the year or two following a strong peak, but decreased to similar abundances to fished areas before the next peak. Snapper length frequency distribution significantly differed between SZ and both fished management types, with more larger snapper within SZ including a higher proportion (58%) that were legal-sized (>25.7 cm FL). HPZ and Outside did not significantly differ from each other, and were dominated by individuals below legal size. Overall, SZ's have positively influenced abundance and length of snapper on these subtropical rocky reefs.

## Introduction

With the implementation of marine protected areas globally, there is increasing need to assess the ecological changes resulting from such spatial management arrangements. Overall, no-take areas (marine sanctuaries) can result in increases in the density and/or biomass of target species [1–3], but this response can be highly variable spatially and temporally due to inherent variability in physical, environmental, ecological and social factors [4–8]. Additionally, evaluating the effects of marine sanctuaries can be difficult as abundance data is often over-dispersed or zero-inflated reflecting biotic patterns, combined with sampling constraints in the marine environment [9]. However, key target species for fishers sampled at suitable scales can be important indicators on which to examine marine sanctuary effects [9]. A significant response to protection on abundance of targeted species can occur over a range of time-scales, but often within the decadal scale [6, 7, 10], a time-scale relevant to applied and adaptive management [8, 11]. Therefore, a spatial comparison around the decadal time-scale is useful for appraisal of management arrangements [12], although increases of target species can occur over longer periods [13–15].

Unlike marine sanctuaries, there may be no differences in abundance between partial protected areas, which are areas still fished by some methods (e.g. fishing-allowed zones in multiple-use marine parks), and adjacent fished areas outside marine parks where only fishing input and output regulations apply (e.g. bag and size limits, gear restrictions) [16, 17]. Likewise, abundances may be similar between marine park zones with different levels of partial protection (e.g. differing restrictions in the types of fishing gears allowed, although both still fished) [18]. This indicates that a response gradient decreasing from marine sanctuaries to partial protected areas and then to fished areas should not necessarily be expected, although there is potential that proximity to a marine sanctuary may increase abundance and/or size relative to areas further away [19]. This proximal increase may occur through movement of fish with a home range greater than a marine sanctuary or through density dependent interactions [19–21]. However, this movement can be spatially constrained with distance from a sanctuary boundary [22, 23], and will depend strongly on the taxa [24] and habitat continuity across zone boundaries [25].

A species often found to have higher abundances within no-take areas compared to adjacent fished areas is snapper (*Chrysophrys auratus*) [9, 26, 27], an opportunistic and generalist predator in the family Sparidae. Snapper is found in temperate and subtropical continental shelf waters of southern Australia and New Zealand, with a sister species in Japan and Indonesia [28–30]. In Australia, adult snapper prefer rocky reef habitat at depths of 20 to 200 m, while juvenile snapper are most abundant in shallower inshore habitats [28, 31]. Snapper are one of the most important target species in the subtropical and temperate waters of the east coast of Australia [32–34] with a long history of exploitation. They are considered growth overfished in NSW and fishing mortality is estimated to be three times that of natural mortality [34, 35]. In NSW the highest reported commercial landings traditionally come from a subtropical region that includes the Solitary Islands Marine Park (SIMP) [32].

The 720 km^2^ SIMP includes marine sanctuaries (Sanctuary Zones: SZ) where fishing is not permitted, and partially protected areas (Habitat Protection Zones: HPZ) where recreational fishing and various types of commercial fishing (e.g. fish trapping, line fishing) are permitted. Snapper is the most-targeted reef-associated fish species in the SIMP by both commercial (trapping and line fishing) and recreational fishers (line fishing) [36, 37]. The SIMP region is important for snapper as it contains extensive areas of rocky reef on the inner continental shelf [38], and whilst snapper in the SIMP show higher abundance in depths greater than 25 m [39], they also occur on shallower reefs in the region [40].

This study compares relative abundance and length of snapper on rocky reefs within three management types: ‘no take’ areas (SZ), partial protected areas (HPZ), and areas outside (Outside) the SIMP over a decadal time-scale. The hypotheses tested are: 1) that the relative abundance of snapper will initially be similar within the three management types, and that within a decade, snapper abundance will be higher in marine sanctuary (SZ) compared to fished areas; 2) that a gradient in response across management types will occur within a decade with abundance in HPZ increasing relative to Outside; 3) that similar patterns to the previous hypotheses will be detected more broadly on deeper reef around the 10 year mark; and 4) that the length distribution of snapper will be larger in SZ after a decade of protection.

## Methods

### Sampling design

**Fig 1:**
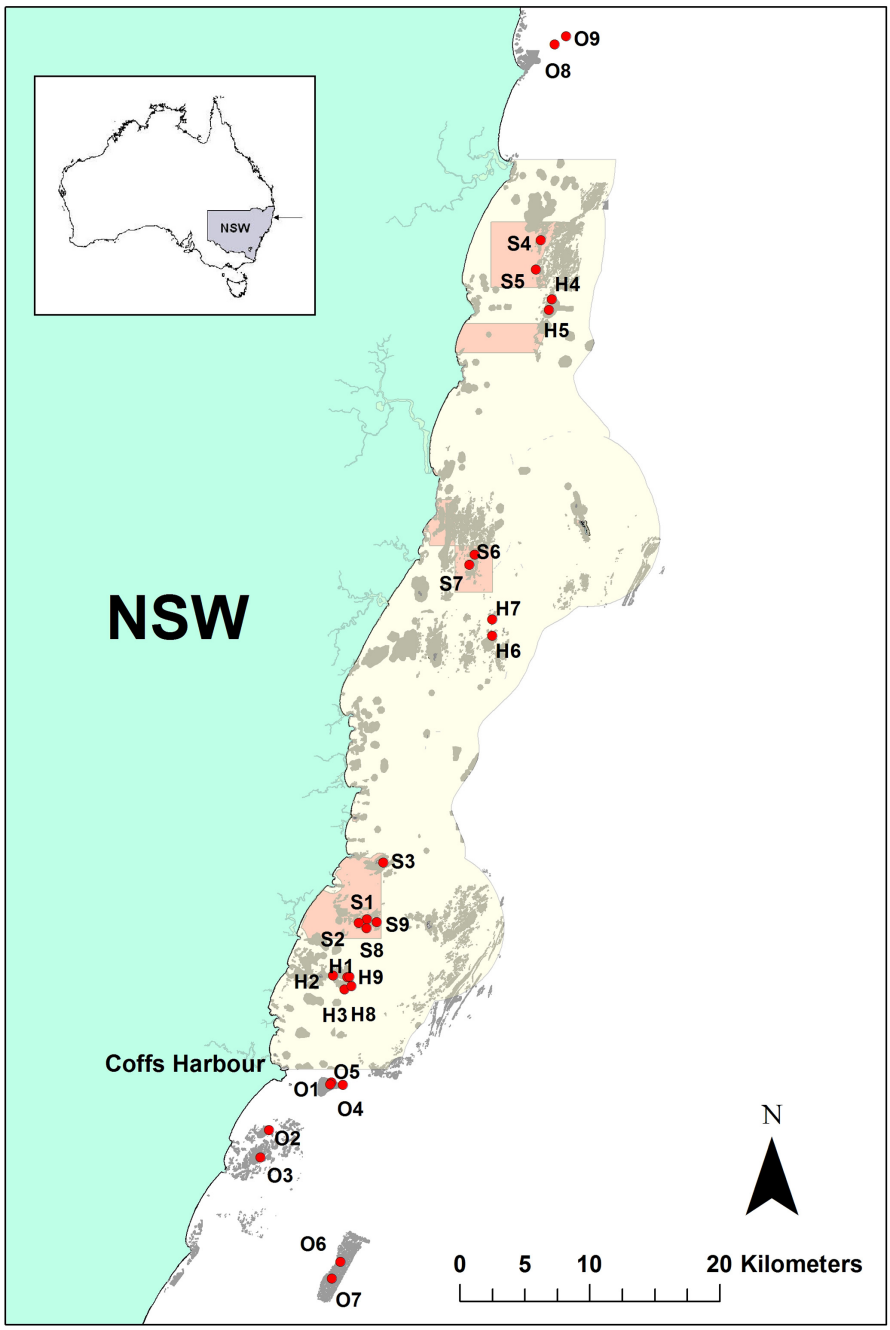
Location of BRUV sites surveyed across three management types (SZ, HPZ, Outside) within and adjacent to the Solitary Islands Marine Park. Sites: S = SZ, H = HPZ, O = Outside. S1–S3, H1–H3, O1–O3 = temporal monitoring sites 2002–2014. S4–S9, H4–H9, O4–O9 = spatial assessment. Pink area = ‘no take’ area (sanctuary zone), yellow = other areas of the marine park where fishing is permitted, including HPZ.

Sampling was conducted within Sanctuary Zones (SZ) and Habitat Protection Zones (HPZ) within the SIMP, and on reefs adjacent to the marine park, defined as Outside (Fig 1) using Baited Remote Underwater Video (BRUV). All sampling was conducted during the Austral winter (June to August).

Sampling was conducted annually from 2002 to 2014 (except 2012) on shallow reef in the 15–22 m depth range. Three sites per management type (Sites S1–S3, H1–H3, O1–O3) in each year were surveyed (12 years in total) with 3 replicate BRUV units deployed for 30 minutes [41] at each site (= totalling 324 deployments). The replicate BRUVs at each site were generally deployed within 5 minutes of each other with at least 200 m distance between them for independence of individual deployments [40]. Shallow rocky reef sites were haphazardly selected from maps derived from single-beam echo soundings around each general reef area with at least 600 m between sites.

Sampling was more-broadly conducted across and adjacent to the SIMP over the three management types in 2010 and 2011, a decade after these sanctuary zones were established, on deeper rocky reef in the 25–40 m depth range. An additional 6 sites were surveyed per management type (although two of the Outside sites were not surveyed in 2010). At each site 4 replicate BRUV units were deployed for 30 minutes each (= 64 deployments in 2010, 72 deployments in 2011). These 18 spatially-broader and deeper sites were selected using maps of rocky reef habitats digitised in ArcGIS from bathymetry and backscatter layers derived from interferometric sidescan sonar surveys [38]. Where swath acoustic coverage was not available (Sites O8, O9), a combination of Maritime chart information and GPS plotter and single-beam echo-soundings were used to select suitable rocky reef in the survey depth range.

While some studies have found no SZ effects on abundance of fishes but have detected increased length and biomass within MPAs [42] there can be observer error resulting in lower precision and power to detect differences based on length [43, 44]. A primary advantage of stereo-BRUVs is to provide accurate length estimates with high precision, enabling comparisons of fish lengths and biomass [45]. Stereo-BRUVs can achieve similar size compositions to those obtainable by line-fishing [46], but without the need for capturing and potentially harming individual animals, an important consideration when sampling in sanctuaries. Stereo-BRUVs were used from 2011 on, including a restricted survey in 2012 at sites S8, S9, H8, H9, O6 and O7. All length data from 2011 to 2014 were merged to provide a balanced (SZ = 59; HPZ = 59; Outside = 59 deployments) length comparison between management types, after a decade of SZ protection.

### Field methods and video analysis

Each BRUV unit consisted of a video camera with wide-angle lens in an underwater housing, an attachment frame, a bait-pole with bait, and a rope and float system linking the BRUV to the surface [39]. Camera technology improved from 2002 until 2014, with original Digital-8 tape video cameras replaced by mini-DVD tapes in 2008, and by digital Canon High Definition video cameras in 2011. Stereo-BRUVs using a dual video camera system were utilised in 2011 to 2014. To attract fish to a viewing area in front of each camera, bait consisted of approximately1 kg of mashed pilchard (*Sardinops neopilchardus*), contained in a plastic mesh bait bag and attached to the end of a bait-pole at a distance of 1.5 m from the lens [47]. Each housing and bait-pole was bolted to a steel frame so that fish could be viewed in a horizontal orientation to the benthos. The tapes were analysed by three authors (HM, PS, AS) with BRUV’s expertise. From each deployment, fish were identified and the maximum number (MaxN) of individual snapper observed in a video frame at any one point of time during the 30-min sample, were recorded. MaxN is a comparative measure of relative abundance that removes any risk of recounting the same individual when determining abundance [48].

Fish were measured from the 2011 to 2014 videos using stereo photogrammetry in EventMeasure (SeaGIS Pty. Ltd.) [43, 49]. The stereo cameras were calibrated using methods previously detailed [50]. Video files were synchronised using a spinning diode that was fixed in front of the camera, enabling frames from each camera to be matched. Fork lengths of snapper were determined around MaxN, as per other stereo-BRUV studies [51], to avoid multiple measurements of the same individuals which would bias the length average.

### Statistical methods

Permutational Multivariate Analysis-of-Variance (PERMANOVA) in PRIMER 6 [52, 53] was used to test for significant differences in snapper abundance (MaxN) for these temporal and spatial data. For the temporal survey a three-factor nested design was used, with Management type (M) as a fixed orthogonal factor, Year (Y) as a random orthogonal factor and Site (S) as a random factor nested in ‘management type’. Homogeneity of variance (or dispersion) was tested using PERMDISP and was highly significant from homogeneity of variance (*P* = 0.0002). Transformed data (log(x+1)) were not significant (*P* = 0.053). Euclidean distance was used as the similarity measure to generate a resemblance matrix of centroids from the transformed univariate data. PERMANOVA analyses were run using type III sums of squares with 9999 permutations, and pair-wise comparisons were undertaken for ‘management type’ if results were significant.

If the three management types were significantly different, their responses were compared by year. These responses were calculated as the log ratio of mean MaxN in sanctuary in each year compared to that in each of the fished zones [1, 3, 54]. If the two ‘fished’ management types were similar, further pairwise comparisons were undertaken to compare them in each year. If HPZ and Outside were similar overall and within each year, they were pooled to provide a ‘fished’ versus ‘no take’ log response ratio by year comparison. The proportion of deployments with snapper present was also visually compared between the three management types over the 12 years, to indicate if there were spatial changes in snapper occurrence.

For the broad-scale spatial comparison, PERMANOVA was used to test across management types, years and sites (three-way nested design) with the same methodology as the temporal comparison. Homogeneity of variance was significant (management type: P = 0.022) until square root transformed (P = 0.5). Snapper at higher density could potentially be expected to appear more rapidly in the BRUV field of view than at lower density, although this will be influenced by their behaviour and spread across a reef (e.g. aggregated or even). Therefore differences in Time at First Sighting (TFS) were compared with PERMDISP and PERMANOVA as above, using a 2-way design with M (fixed) and Year (random). Data were pooled by site, due to a maximum of only one TFS record for snapper per deployment on those replicates where snapper was recorded.

The influence of depth on management response was tested between shallower and deeper sites using a 3-way PERMANOVA with Depth (D: shallow reef, intermediate-depth reef) as a fixed orthogonal factor, Management type (M) as a fixed orthogonal factor, and Year (Y: 2010, 2011) as a random orthogonal factor. Homogeneity of variance remained significant following transformations, with Log(X+1) the least significant (MDY: P = 0.031) so this transformation was applied.

Fork lengths for snapper in 2011 to 2014 were aggregated within 2 cm bins by management type to generate a length frequency distribution for visual comparison between the three management types over this period, which is 9 to 12 years after the zoning plan was established. Differences in length distributions between management types were compared using the Kolmogorov-Smirnov test. This test calculates the maximum distance between two cumulative distributions (test statistic D), and determines a corresponding P value. Three pairwise tests were conducted (SZ vs HPZ, SZ vs Outside, HPZ vs Outside) with significant P values (<0.05) indicating differences in their relative cumulative length distributions. Differences between years by management type were not compared due to too few individual measurements in 2012, 2013 and 2014. The proportions of snapper above legal minimum size in NSW (~25.7 cm FL) were determined for the three management types and compared between these using a 1-way PERMANOVA with two variables (< 25.7 cm, > = 25.7 cm) and two values (1 = yes, 0 = no) for each length measure. The proportions above the estimated 50% maturity length for northern NSW (21.8 cm) were also determined.

### Ethics statement

This research was undertaken under NSW DPI Fisheries Scientific Permit P01/0059(A)-2.0. This project was undertaken in accordance with NSW DPI Animal Care and Ethics permit ACEC10/09. No fish were collected or harmed during this research.

## Results

A total of 1,081 snapper (summed MaxN) were recorded from 484 BRUV deployments in total (SZ: 164; HPZ: 164; Outside: 156 deployments). Overall, 580 snapper were recorded from SZ, 265 from HPZ and 236 from Outside.

### Comparison of abundance (MaxN) over years on 15 to 25 m deep reef

**Fig 2:**
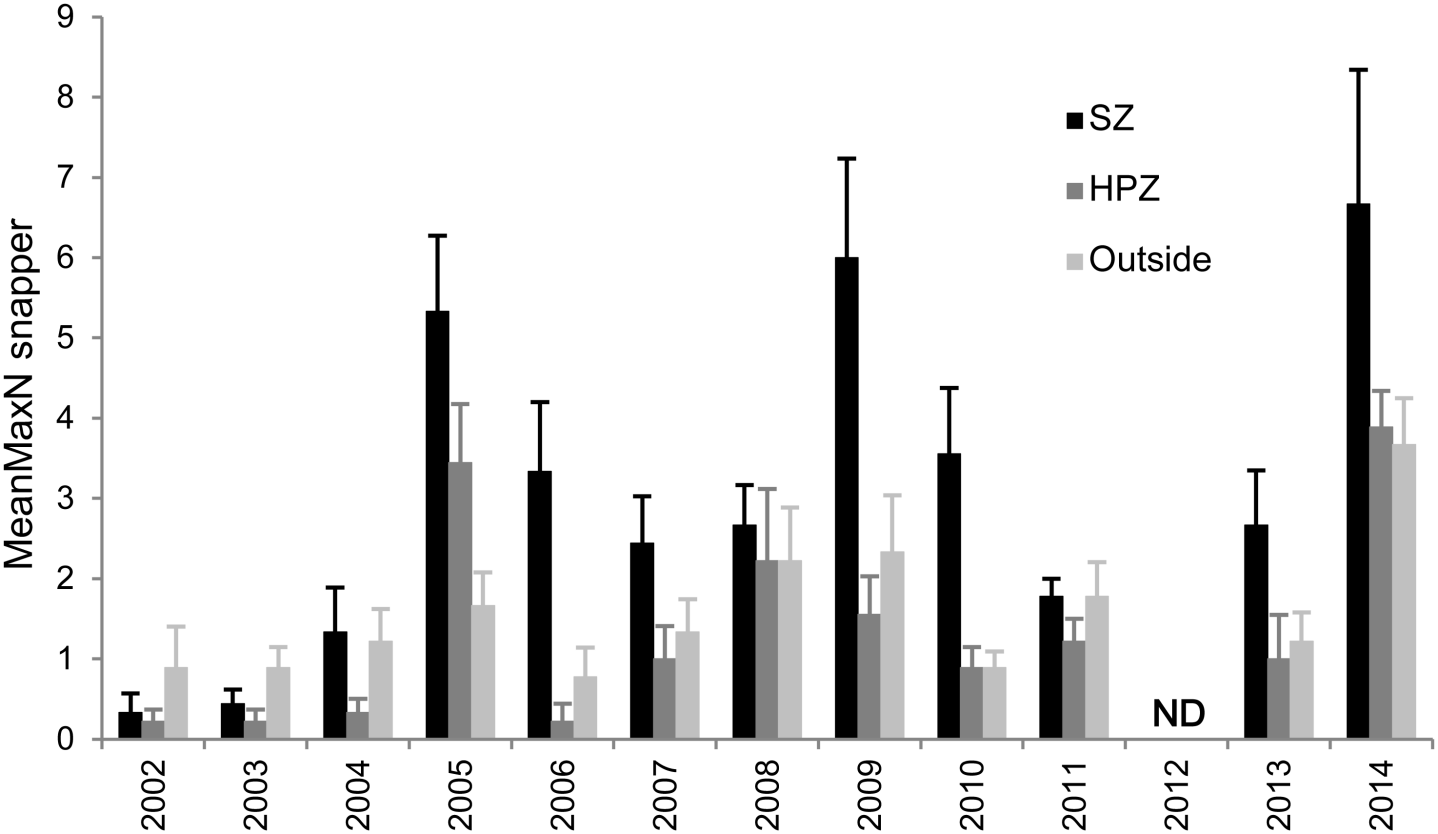
Average abundance (mean MaxN) of snapper on shallow reef by year and by management type. SZ = Sanctuary Zone, HPZ = Habitat Protection Zone, Outside = outside the marine park. Standard error bars. ND = no data.

**Fig 3:**
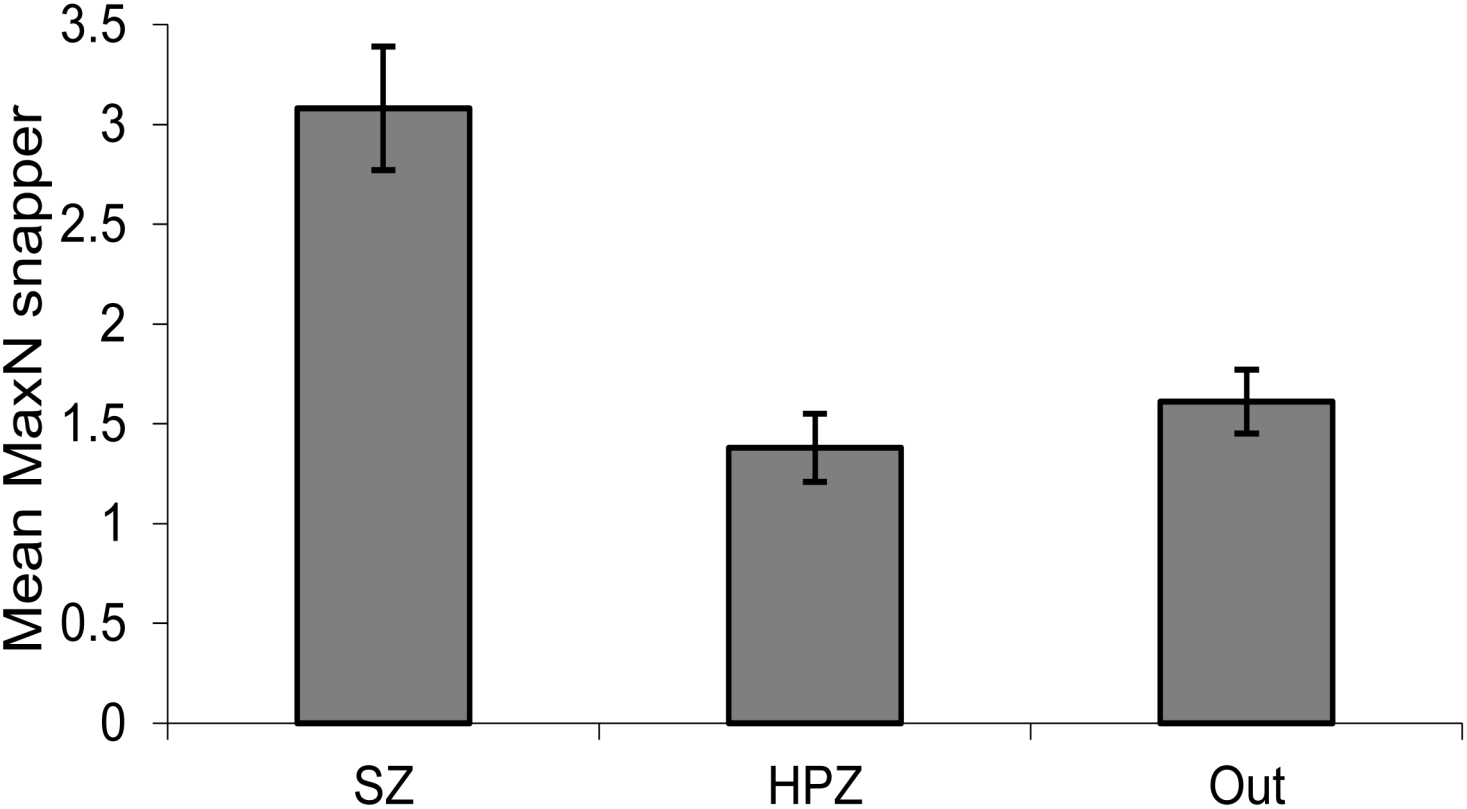
Average abundance (mean MaxN) of snapper on shallow reef by management type. SZ = Sanctuary Zone, HPZ = Habitat Protection Zone, Outside = outside the marine park. Averaged over the 11 survey periods. Standard error bars.

Following establishment of marine sanctuaries (SZ) in 2002 there was a rapid increase in snapper, particularly in 2005 after the first three years of protection (Fig 2). Abundance varied considerably between years, but in most years following 2005 there were more snapper in SZ than in HPZ or Outside. Abundance declined rapidly in fished areas in the year following this peak (2006), but declined more slowly in SZ over two to three years. This pattern of peak and decline was subsequently repeated 2009 to 2011. There were significant differences for the main terms: Management type (M) and Year (Y) and also for their interaction (M×Y) (Table 1, Fig 2). Overall, there were more snapper after the first two years, with peaks in 2005, 2009 and 2014. Pairwise comparisons (for M) show SZ is highly significantly different from both HPZ and Outside, which are similar to each other (Table 1, Fig 3).

**Table 1:** Results of Permutational Analysis of Variance (PERMANOVA) for relative abundance of snapper (MaxN) across the factors.

Source	df	SS	MS	Pseudo-F	P(perm)	Perms	Significance
M	2	15.047	7.5234	9.2458	**0.0002**	9934	***
Y	11	49.003	4.4549	19.522	**0.0001**	9915	***
S(M)	6	1.7142	0.28569	1.252	0.2887	9937	
M×Y	22	12.159	0.5527	2.422	**0.0031**	9926	**
Y×S(M)	66	15.061	0.2282	0.8583	0.7666	9843	
Res	216	57.428	0.26587				
Total	323	150.41					
**Groups**				**t**	**P(perm)**	**Perms**	**Difference**
SZ, HPZ				3.9273	**0.0003**	9948	SZ >>> HPZ
SZ, Out				2.8595	**0.0027**	9953	SZ >> Outside
HPZ, Out				1.4937	0.1085	9941	HPZ = Outside

M = management type; Y = year; S = site (nested in M). SZ = Sanctuary Zone, HPZ = Habitat Protection Zone; Out = Outside. Significant P values (<0.05) shown in bold.

*** P < 0.001,

** P < 0.01,

* P < 0.05.

Pairwise comparisons for Management (Factor: M).

**Fig 4:**
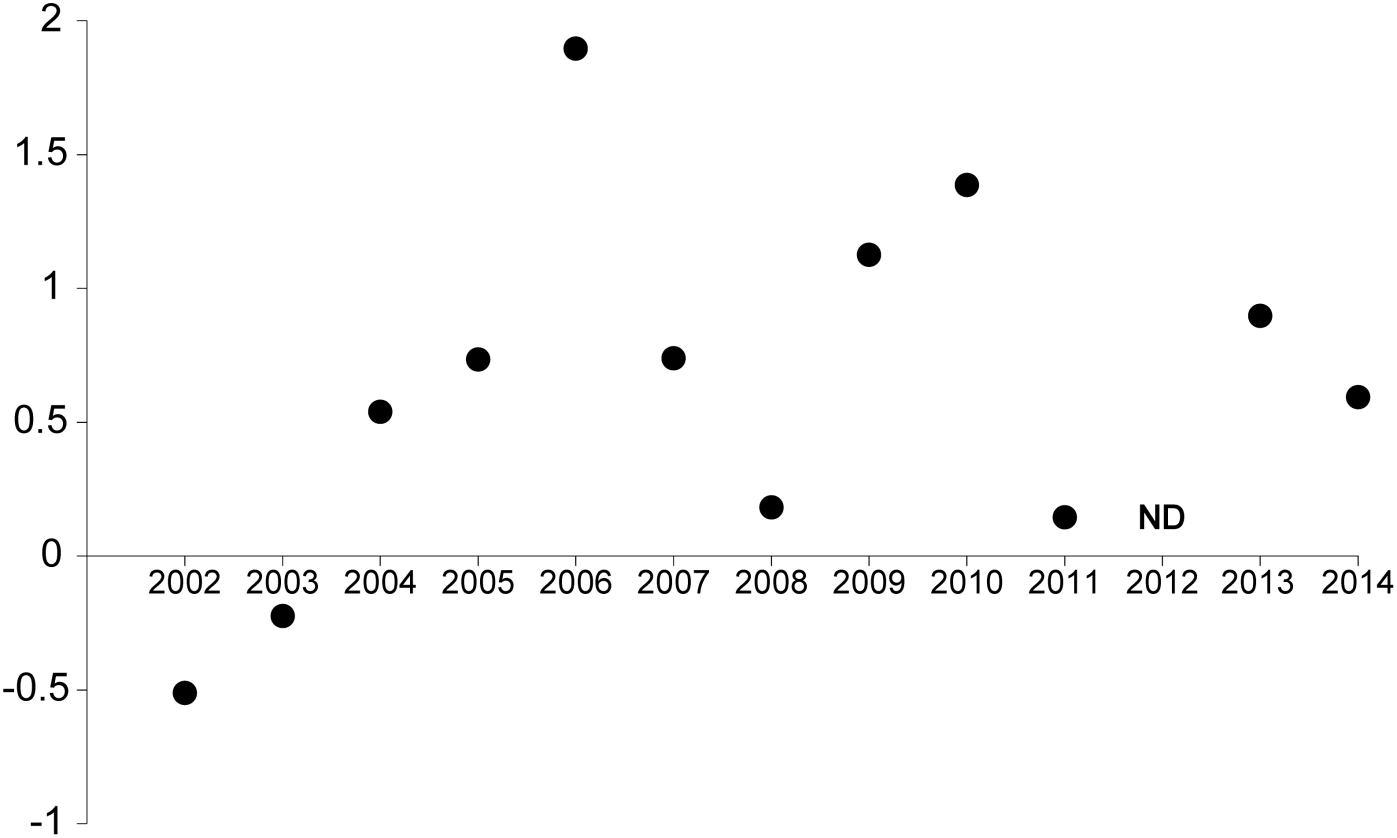
Log response ratio of snapper on shallow reef: annual averages of snapper in SZ vs fished zones (annual averages of HPZ + Outside). SZ = Sanctuary Zone, HPZ = Habitat Protection Zone, Outside = outside the marine park. ND = no data.

Given HPZ and Outside were similar in abundance (Fig 3, Table 1) and not significantly different in all 12 years (from pairwise comparisons for each year), they were pooled for comparison with the SZ response to provide a ‘fished’ versus ‘no take’ comparison. This log response ratio of annual average snapper in SZ to average annual snapper in fished areas (HPZ + Outside) indicates that after the first two years (2002, 2003), a positive response occurs and then remains positive in most years. In 2004 the relative response is mainly due to increase in SZ but not HPZ. This response ratio is considered strong in most years, but fluctuates. The strongest response is in the years 2006 and 2010, which immediately follow two peaks in abundance, which occur in 2005 and 2009. The smallest response (excluding 2002 and 2003) is in 2008 and 2011, which are three and two years respectively after the peak abundance years (Fig 4). Snapper abundance is very similar among the three management types in these two years at these shallower reef sites.

**Fig 5:**
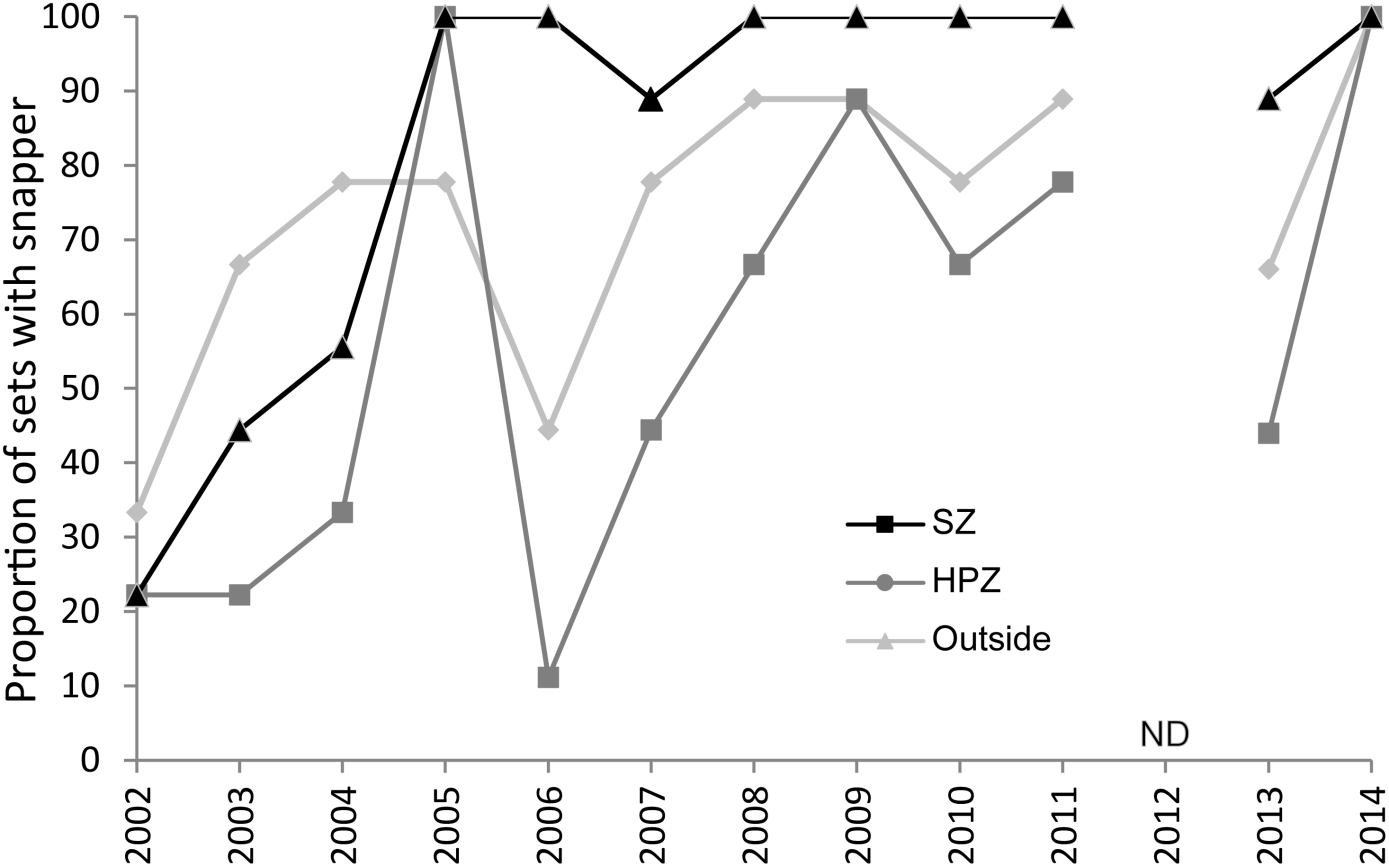
Proportion of shallow reef deployments (%) with snapper within each management type by year. SZ—Sanctuary Zone; HPZ = Habitat Protection Zone; Outside = outside the marine park. ND = no data.

Overall, snapper were more evenly distributed spatially within SZ than HPZ and Outside over time, as indicated by the proportion occurring on deployments (Fig 5). In 2002, when the marine sanctuaries were only just being established, snapper occurred on ~20% of replicates inside the SIMP (SZ and HPZ) and ~30% outside. This increased rapidly in the following three years, with both SZ and HPZ having snapper on all deployments in 2005, although not on all Outside deployments. Since 2005 most (>90%) of deployments in SZ had snapper, whereas this was more variable in the other two management types (Fig 5).

### Broader-scale spatial comparison of abundance (MaxN) in 2010 and 2011 on deeper reef

A total MaxN of 385 snapper were recorded on the 136 deployments in 2010 and 2011 on the deeper (25–40 m) sites used to assess the spatially broader pattern. In 2011, when the sampling design was fully-balanced, 139 snapper were recorded in SZ, 51 in HPZ, and 40 Outside.

**Fig 6:**
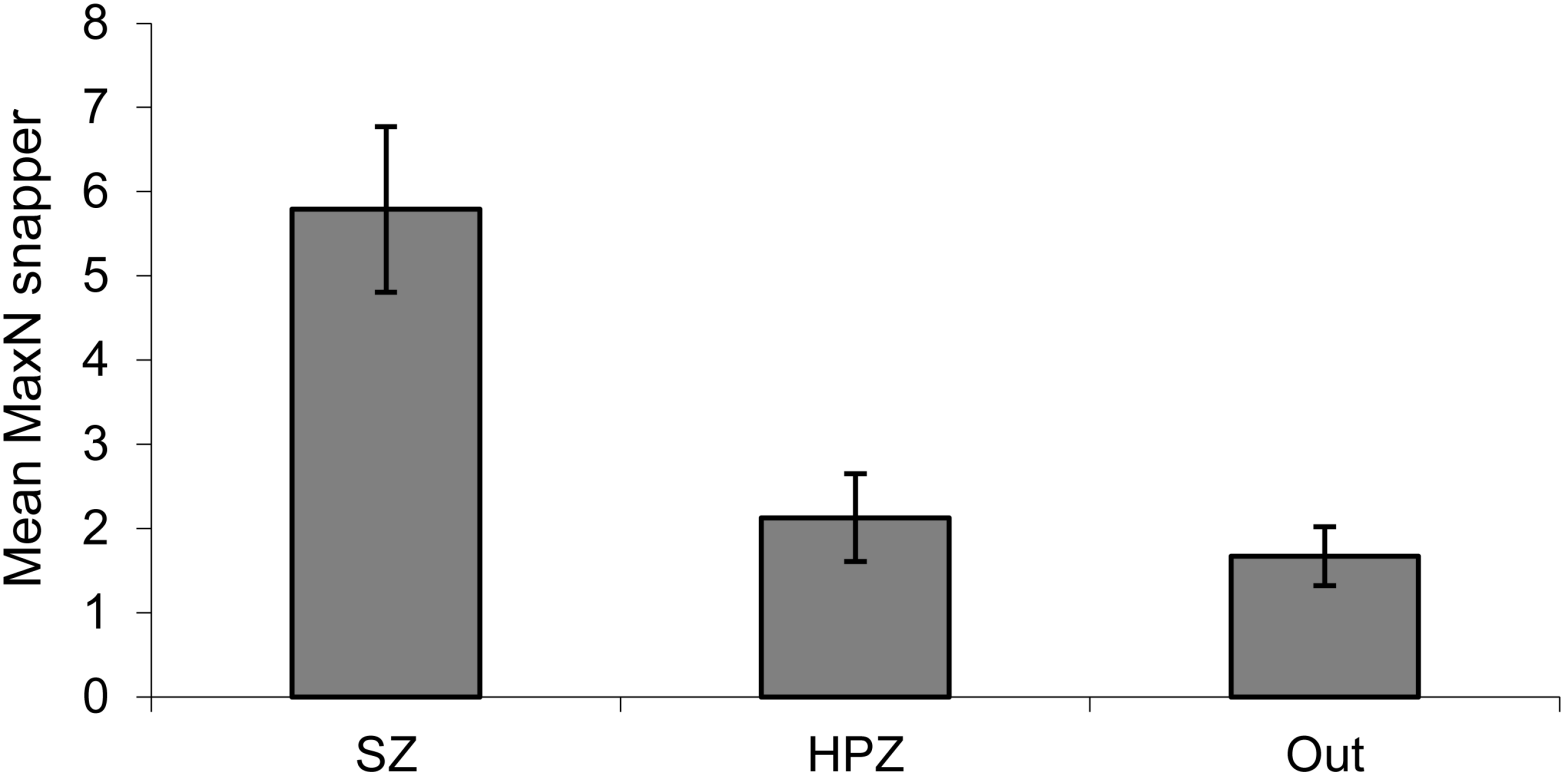
Mean MaxN by management type on deeper reef. SZ = Sanctuary Zone, HPZ = Habitat Protection Zone; Out = Outside. Error bars = SE.

From PERMANOVA, the abundance (MaxN) of snapper was significantly different between management types (Table 2, Fig 6). From pair-wise comparisons for management type, SZ was significantly different from both HPZ and Outside, which were similar to each other (Table 2). The interaction of site (nested in management type) and year was also significant (Table 2). Snapper were more abundant in SZ in both 2010 (1.8× HPZ, 2.5× Outside) and 2011 (2× HPZ, 3.5× Outside). Examination of site by year averages showed that some sites in SZ had higher MaxN than others and this was not always consistent between years, which appears to have driven this significant interaction. One HPZ site had higher abundance than other HPZ sites and some SZ sites in both years, whereas all Outside sites were predominantly lower relative to SZ sites.

**Table 2:** Results of Permutational Analysis of Variance (PERMANOVA) for relative abundance of snapper (MaxN) across the factors.

Source	df	SS	MS	Pseudo-F	P(perm)	Perms	Significance
M	2	28.179	14.09	5.4178	**0.0087**	9959	**
Y	1	2.5114	2.5114	2.2889	0.1538	9823	
S(M)	15	36.14	2.4094	2.1959	0.0803	9945	
M×Y	2	1.0634	0.5317	0.4846	0.6269	9956	
Y×S(M)	13	14.263	1.0972	2.0055	**0.0298**	9935	*
Res	102	55.804	0.54709				
Total	135	138.01					
**Groups**				**t**	**P(perm)**	**Perms**	**Difference**
SZ, HPZ				2.5694	**0.0191**	9961	SZ > HPZ
SZ, Out				2.9124	**0.0124**	9964	SZ > Outside
HPZ, Out				0.42488	0.9626	9967	HPZ = Outside

M = management type; Y = year; S = site (nested in M). SZ = Sanctuary Zone, HPZ = Habitat Protection Zone; Out = Outside. Significant P values (<0.05) shown in bold.

*** P < 0.001,

** P < 0.01,

* P < 0.05.

Pairwise comparisons for Management (Factor: M).

Snapper also occurred on a higher proportion of BRUV replicates in SZ in both 2010 (92%) and 2011 (100%) compared with HPZ (54%, 71%) and Outside (71%, 62%). We also examined Time to First Sighting (TFS), however this metric did not significantly differ between management types. The average TFS in both years for all management types was less than 10 minutes. The proportion of TFS records within 2 minute time bins was concentrated within the first six minutes for all three management types.

### Comparison of abundance (MaxN) between shallower and deeper reef in 2010 and 2011

From PERMANOVA, there was no significant difference in the abundance of snapper detected for the three factors and their interactions. Most were strongly non-significant (P>0.25), although the interaction between shallower and deeper sites in the three management types for the two years examined (YMD) and management type (M) were more marginal (P = 0.067, P = 0.070, respectively). This was partly-driven by the 2011 shallow sites having a similar average-MaxN in all three management types (Fig 2). At the deeper sites, snapper were more abundant in SZ in both 2010 (1.8× HPZ, 2.5× Outside) and 2011 (2× HPZ, 3.5× Outside), whereas the SZ response was more variable on shallower reef, being larger in 2010 (4× HPZ, 4× Outside) and smaller in 2011 (1.5× HPZ, 1× O).

### Comparison of lengths between management type (2011–2014)

From the 177 deployments (2011–2014), a total of 431 snapper were measured for Fork Length (FL). More individuals were measured in SZ (250) than HPZ (109) and Outside (72) due to the higher total MaxN in SZ. Both average FL and maximum FL were greater in SZ (Table 3). The cumulative length distribution significantly differed between SZ and the other two management types, which did not differ among each other (Table 3), even excluding the large SZ outlier snapper (66.9 cm FL).

**Table 3:** Maximum, minimum and average Fork Lengths (±SE) by management type.

	SZ	HPZ	Outside
Maximum (cm)	**66.9**	38.3	36.8
Minimum (cm)	18.9	15.3	17.5
Average (cm) (SE)	**27.4** (3.4)	22.9 (3.5)	23.8 (3.9)
N (number of measurements)	250	109	72
**Kolmogorov-Smirnov test**	**D statistic**	**P**	
SZ vs HPZ	0.4499	**0.000**	
SZ vs Out	0.3337	**0.000**	
HPZ vs Out	0.1682	0.155	

Results of Kolmogorov-Smirnov test comparing relative cumulative length distribution between management type. D statistic = maximum difference between cumulative distributions with corresponding P value. Significant differences (P< 0.05) are bolded. SZ = sanctuary zone; HPZ = Habitat Protection Zone; Out = Outside.

**Fig 7:**
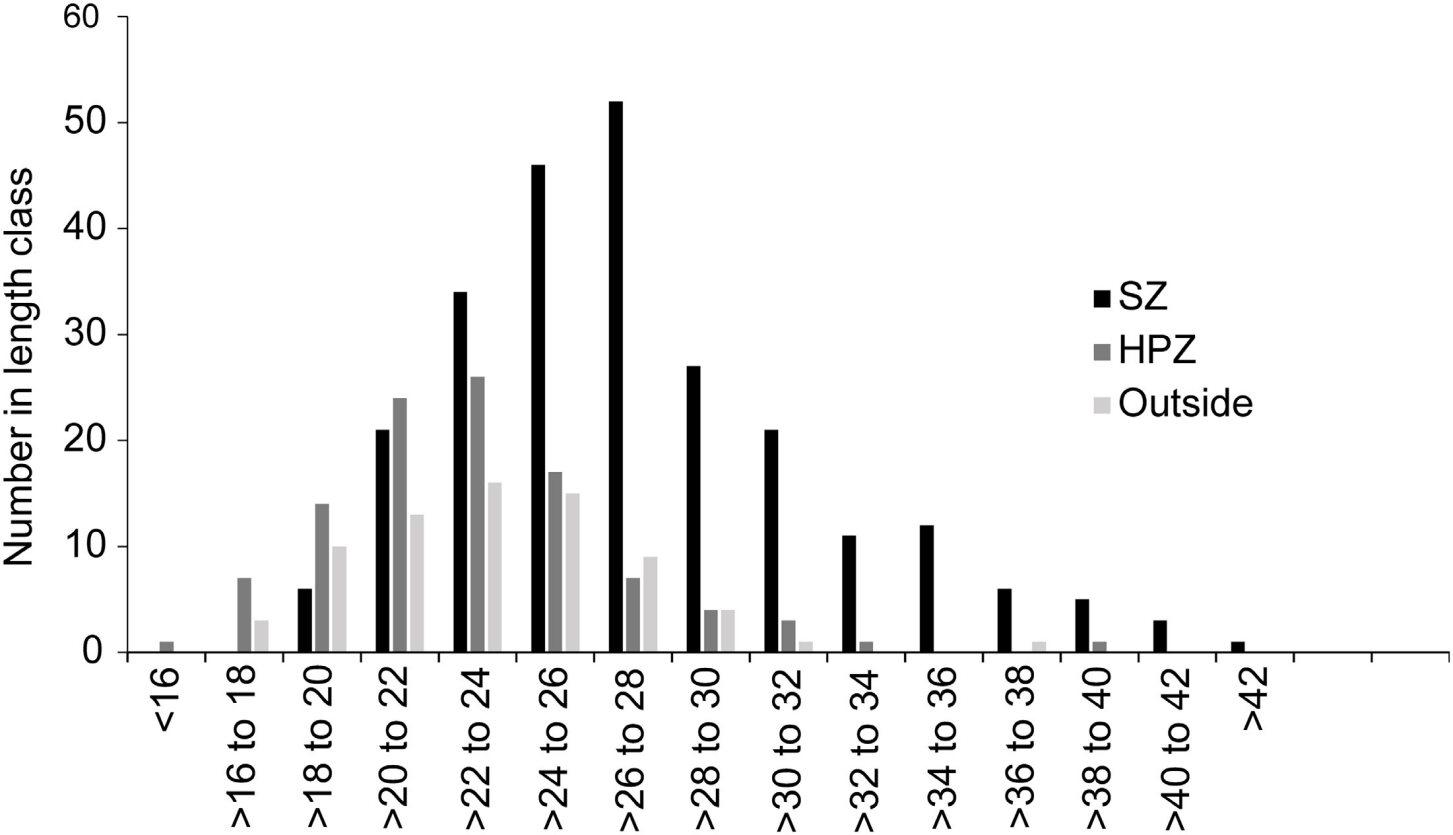
Length frequency (fork length) distribution for 2011–2014 by management type in 2 cm bins. SZ = Sanctuary Zone, N = 249; HPZ = Habitat Protection Zone, N = 109; Outside = Outside the marine park, N = 72 snapper.

Snapper had a broader length frequency distribution in SZ than HPZ and Outside, with larger fish up to 42 cm FL, and one large outlier individual (66.9 cm) (Fig 7). The minimum legal size of snapper in NSW is 30 cm Total Length (TL), which is approximately 25.7 cm FL [55], and a tight relationship (R^2^ = 0.9935) between TL and FL has been confirmed for NSW (NSW Department of Primary Industries, unpublished data). From the stereo-BRUV measurements the proportion of snapper above the estimated minimum legal fork length was higher in SZ (58%) compared with HPZ (17%) and Outside (29%). PERMANOVA of the proportions of individuals above and below 25.7 cm FL within each management type, indicates that SZ was very different from both HPZ (P = 0.0001) and Outside (P = 0.0002), which were also different from each other (P = 0.0299).

Approximately 50% of snapper were found to mature at about 21.8 cm FL in northern NSW during fisheries surveys in 2008, which was a reduction from that previously determined in the 1980’s (28 cm FL) [35]. From the stereo-BRUV measurements, the proportion of snapper above 21.8 cm fork length was higher in SZ (90%) compared with HPZ (59%) and Outside (72%).

## Discussion

Overall, there has been a significant response in snapper abundance and length within marine sanctuaries in the subtropical SIMP at the decadal scale. Since 2002, the abundance and occurrence of snapper on the reefs in the SIMP region, both within and outside of sanctuaries, has generally increased, although with considerable variation between years and sites. An increase occurred rapidly, within the first three years following the establishment of sanctuary zones (SZ’s) in 2002, with a sevenfold increase in average MaxN (all sites combined) from 2002 to 2005. Overall, the largest increase in abundance has been within SZ. Rapid changes in target species abundance in marine sanctuaries relative to fished areas have also been detected in other studies [7], including strong snapper responses [9, 26, 27, 56].

After the first three years following establishment of these SIMP zones, the response ratios between SZ and fished areas for most of the years in this study were considered strong (cf. >0.69 in [57]). In particular, snapper abundance within SZ was enhanced during years of peak abundance (e.g. 2005, 2009) and sustained over following years, and the strongest response ratios were in the years immediately following peaks in SZ. However, on shallower SZ sites snapper generally declined to similar levels to HPZ and Outside by the second or third year after each SZ peak (e.g. 2008, 2011). These peaks may be partly-influenced by recruitment variability. There is evidence that snapper in southern Australia have peaks in recruitment approximately every four years [58], and strong variation between years in the abundance of snapper in New Zealand marine sanctuaries were also considered to be influenced by recruitment peaks [9].

This variability may also be partly-influenced by movement patterns. It is likely some snapper remain within these SZ boundaries within and between years, as tagging studies further north in Moreton Bay [59] and further south in central NSW (NSW DPI unpub. data.) indicate that most snapper are resident at a scale less than a few km’s, although there are also some large-scale (e.g. 100’s of km’s) movements [59, 60], However, the scale of residency may be regionally variable depending on the spatial configuration of their preferred continental shelf habitats, and a proportion may have a home range larger than the area covered by marine sanctuary [61]. Anecdotally, there may be seasonal inshore-offshore movements of snapper in the SIMP (NSW DPI unpub. data.), probably in relation to winter peak spawning activity in northern NSW. Similar inshore-offshore movement patterns in snapper have been suggested in temperate waters in relation to spawning [26, 30, 62]. Interestingly, even if there is substantial snapper movement across SZ boundaries in the SIMP, increased abundance has still been largest in SZ. This would support broader meta-analyses findings indicating that commercially exploited species with larger home ranges can still show strong marine sanctuary benefits [63]. However, further work is required on seasonal and fine-scale movement of snapper in this region to examine this more-fully.

If there is substantial movement of snapper across SZ boundaries, then fishing mortality may also have partly-influenced the strong temporal variability. Outside of sanctuaries, snapper are likely to be subjected to high levels of fishing mortality over the scale of a few years, as evidenced by age truncation in the NSW population due to exploitation, with few snapper older than 5 years [64]. Snapper is one of the most important species in both the commercial trap and line and recreational fisheries in northern NSW [32, 37], with a similar harvest from each in recent times within NSW (~200 tonnes). Snapper is growth overfished in NSW, with a fishing mortality three times that of natural mortality, with commercial landings declining 75% since the 1980’s [34].

Snapper have evolved a life history with potential longevity of 40 years, but maturation at a relatively young age (e.g. 50% mature at ~1.7 years in northern NSW) [35], a strategy that can provide for a long reproductive life and assist population persistence through periods unfavourable to recruitment [64]. However, age truncation in snapper is a management concern given older fish are likely to be important to population resilience [64], with larger snapper more likely to be mature and have higher fecundity [35] and higher quality of eggs [65]. From measurements in 2011 to 2014 many of the snapper in this study are probably young (<3 yr), with most probably <5 yr [29, 32], although some may have been considerably older given a weak relationship between size and age [34]. However the larger fish, especially those above the legal size limit in NSW, were predominantly recorded in SZ.

If snapper survivorship is improved by marine sanctuaries and the fish are reaching larger sizes and ages, as our findings indicate, this may contribute at a local scale towards rebuilding abundance and size structure in the population. This may be enhanced where marine sanctuaries include habitats used by a species across all life-history stages and seasonal distributions [66]. However, the positive effect of marine sanctuaries on the overall snapper population in this region may be constrained by the smaller extent of reef protected in marine sanctuaries at the regional scale, as well as any illegal fishing that may occur [8, 67, 68].

We hypothesised that there would be a gradient of response in snapper abundance from marine sanctuary (SZ) to partial protected area (HPZ) to outside the marine park (Outside) given closer proximity of HPZ sites to SZ and the potential for adult snapper movement between them. However, no difference was found between HPZ and Outside. This was considered a potential outcome given recreational fishing and commercial fish trapping are permitted at both the HPZ and Outside sites, and given findings elsewhere [16, 17]. Additionally, the HPZ sites are not connected by continuous reef to SZ, and are physically separated from SZ by sand habitats over the kilometre scale [25, 69, 70].

As we hypothesised, the findings of the broader spatial scale sampling on deeper reef (25–40 m) a decade after the SZ sites were protected from fishing reflect the positive response in snapper abundance in SZ indicated by the shallower temporal study. Snapper were significantly more abundant in SZ compared to the fished areas on these deeper reefs. This supports the positive influence of SZ on snapper abundance, at a broader scale. Likewise, HPZ and Outside did not significantly differ among each other, also reinforcing the results from the shallower sites. Although we considered there may be a larger response on deeper reefs due to snapper being more abundant generally in the SIMP below 25 m depth [39], this was inconsistent over these two years. Again, further work is required on movement of snapper in this region to examine the possible influence of seasonal migration on these patterns, especially on shallower reef.

Establishing the marine sanctuaries in this study was not the only management strategy undertaken in 2002 likely to influence snapper abundance. Additional to spatial management there was a reduction in commercial fishing effort in 2002 in the SIMP as part of the marine park re-zoning through buy-out of some commercial fishing licences. Also, in 2001 the NSW minimum size limit for snapper increased from 28 cm to 30 cm Total Length. Complementary to spatial management, these input and output controls would have benefitted the overall region, including the reefs in this study [35]. However, in subsequent years commercial fish trapping has increased in the area, and fishing effort is similar to before the buy-out, with snapper still a key target species (DPI unpub. data.). At the same time, there have also been major changes in technology and information over the past two decades that may have increased pressure on snapper [71], and changes in use of coastal areas by an increasing population.

Overall, the marine sanctuaries in this study have resulted in an increase in snapper abundance and length, positively benefitting this highly targeted and socially important species within the SIMP region. More broadly, we have demonstrated the value of decadal-scale datasets for objectively assessing the effects of marine sanctuaries on targeted species such as snapper.

## Supporting Information

S1 Dataset Abundance (MaxN) and Fork length (FL mm) raw data. (XLSX)
